# A Calm Editor

**Published:** 1899-12-02

**Authors:** 


### A Calm Editor.

A curious instance of the attitude of calm serenity adopted by the medical profession in the face of agitating political events is afforded by a paper just to hand, namely, the *South African Medical Journal*, the journal of the South African Medical Association, dated November, 1899. So interested is everybody here in all that has to do with the war now going on that the editorial eye naturally turned to this paper in the hope of finding items of interest to our readers. Yet, after searching it from end to end we cannot find even an allusion to the war, or a single hint that such a thing exists. We do not criticise, we only wonder at the phlegmatic temperament of its editors. To be equal to all occasions is no doubt judicious, but when British blood is being so freely shed for the protection of our colony this calm impassiveness among those on the spot is slightly aggravating.

